# Sex Modulates the Pathological Aging Effect on Caudate Functional Connectivity in Mild Cognitive Impairment

**DOI:** 10.3389/fpsyt.2022.804168

**Published:** 2022-04-05

**Authors:** Zhengshi Yang, Jessica Z. K. Caldwell, Jeffrey L. Cummings, Aaron Ritter, Jefferson W. Kinney, Dietmar Cordes, The Alzheimer's Disease Neuroimaging Initiative (ADNI), Michael Weiner

**Affiliations:** ^1^Cleveland Clinic Lou Ruvo Center for Brain Health, Las Vegas, NV, United States; ^2^Department of Brain Health, University of Nevada Las Vegas, Las Vegas, NV, United States; ^3^Chambers-Grundy Center for Transformative Neuroscience, Department of Brain Health, School of Integrated Health Sciences, University of Nevada Las Vegas, Las Vegas, NV, United States; ^4^Department of Psychology and Neuroscience, University of Colorado, Boulder, CO, United States

**Keywords:** aging effect, caudate, mild cognitive impairment, functional connectivity, Alzheimer's Disease, sex difference

## Abstract

**Purpose:**

To assess the pathological aging effect on caudate functional connectivity among mild cognitive impairment (MCI) participants and examine whether and how sex and amyloid contribute to this process.

**Materials and Methods:**

Two hundred and seventy-seven functional magnetic resonance imaging (fMRI) sessions from 163 cognitive normal (CN) older adults and 309 sessions from 139 participants with MCI were included as the main sample in our analysis. Pearson's correlation was used to characterize the functional connectivity (FC) between caudate nuclei and each brain region, then caudate nodal strength was computed to quantify the overall caudate FC strength. Association analysis between caudate nodal strength and age was carried out in MCI and CN separately using linear mixed effect (LME) model with covariates (education, handedness, sex, Apolipoprotein E4, and intra-subject effect). Analysis of covariance was conducted to investigate sex, amyloid status, and their interaction effects on aging with the fMRI data subset having amyloid status available. LME model was applied to women and men separately within MCI group to evaluate aging effects on caudate nodal strength and each region's connectivity with caudate nuclei. We then evaluated the roles of sex and amyloid status in the associations of neuropsychological scores with age or caudate nodal strength. An independent cohort was used to validate the sex-dependent aging effects in MCI.

**Results:**

The MCI group had significantly stronger age-related increase of caudate nodal strength compared to the CN group. Analyzing women and men separately revealed that the aging effect on caudate nodal strength among MCI participants was significant only for women (left: *P* = 6.23 × 10^−7^, right: *P* = 3.37 × 10^−8^), but not for men (*P* > 0.3 for bilateral caudate nuclei). The aging effects on caudate nodal strength were not significantly mediated by brain amyloid burden. Caudate connectivity with ventral prefrontal cortex substantially contributed to the aging effect on caudate nodal strength in women with MCI. Higher caudate nodal strength is significantly related to worse cognitive performance in women but not in men with MCI.

**Conclusion:**

Sex modulates the pathological aging effects on caudate nodal strength in MCI regardless of amyloid status. Caudate nodal strength may be a sensitive biomarker of pathological aging in women with MCI.

## Introduction

Brain aging is characterized by considerable heterogeneity, including the differences between regions and the variability induced by demographic factors and symptomatic or presymptomatic pathology, such as the presence of brain amyloid. These issues may be especially important for studies of mild cognitive impairment (MCI), known to be a heterogeneous condition (1). The medial temporal lobe memory system and frontostriatal system are well-recognized as two fundamental neural systems supporting episodic memory and executive function (2, 3). While episodic memories are established and maintained by an interplay between the medial temporal lobe and other cortical regions, the aging-related degradation of the frontostriatal system is suggested to be a driving factor of episodic memory decline in older adults (4). Caudate nucleus is of particular interest in the frontostriatal system because caudate function and structure is sensitive to age-related differences between healthy young and old adults. Cortical-caudate functional connectivity was revealed to be less differentiated in older adults compared to younger adults, and age-related differences in caudate function were associated with memory decline in normal aging (5). Task functional magnetic resonance imaging (fMRI) data showed that, in a virtual navigation task, older adults had significant activity in the caudate nucleus while young adults had significant activity in the hippocampus, suggesting that the aging process involves a shift of functional demands from hippocampus toward the caudate nucleus during navigation (6). Structural magnetic resonance imaging (MRI) studies in elderly adults demonstrated the multifaceted role of caudate nucleus with evidence that caudate atrophy is related to various behavioral performances, including slower walking speed (7) and longer action selection time (8). In addition, damage to the caudate nucleus is accompanied by decline in inhibitory processes, executive control, and cognitive speed similar to the effects observed in normal aging (9). Neurodegenerative conditions could lead to the alteration in caudate nucleus, which were mainly reported in Parkinson's disease (10, 11), however, the role of caudate in mild cognitive impairment (MCI) or Alzheimer's dementia (AD) largely remains unknown.

Neurodegenerative conditions can alter the brain trajectory that differs from normal aging. Identifying pathological aging effects in subjects with neurodegenerative disease may provide critical insights into underlying disease mechanisms. Amyloid positive MCI is the prodromal stage that have a higher incidence of AD conversion. Investigating age-related effects on neuronal activity among amyloid positive and amyloid negative MCI patients is potentially critical for developing therapeutic strategies to slow or prevent more severe disease progression. Furthermore, emerging evidence suggests that women differ from men in multiple neurological aspects, including brain function (12), cognitive domains, cognitive decline (13, 14), and effects of amyloid deposition (15). Sex was also found to modulate aging effects on brain atrophy in healthy adults (16). A recent study showed significant sex-modulated aging effects on plasma total tau protein in individuals with subjective memory complaints (17). These findings suggest that sex is at least partially responsible for the clinical and pathological heterogeneity of AD, and it is likely to mediate age-related and amyloid-related degeneration in the brain. A better understanding of sex-specific risk and protective factors in MCI is crucial for developing personalized therapeutic strategies.

In this study, we focused on age-related effects on caudate function by analyzing resting-state functional magnetic resonance imaging (fMRI) data from subjects with normal cognition and subjects with MCI from the Alzheimer's Disease Neuroimaging Initiative (ADNI) project (18). Functional connectivity analysis was first carried out to assess the functional coupling strength of caudate nuclei with individual regions, then graph theoretical analysis was applied to derive a scalar metric, namely nodal strength, to characterize the overall caudate connectivity strength. Linear mixed effect (LME) modeling was utilized to evaluate a sex-dependent association of age with caudate nodal strength and caudate connectivity with individual regions. With the subset of participants with fMRI data and amyloid positron emission tomography (PET) available at the same visit, we assessed the association separately for those with and without amyloid burden. We hypothesized that (1) MCI has a stronger age-related increase in caudate functional connectivity compared to cognitive normal (CN) subjects, and (2) sex, together with brain amyloid, modulates the pathological aging effects on caudate nuclei in MCI. The purpose of this study is to examine whether and how sex and amyloid mediate age-related effects on caudate function in MCI.

## Materials and Methods

### Main Sample

Data used as main sample in this study were de-identified and obtained from the ADNI database in September 2019. The study was approved by each participating ADNI site's local Institutional Review Board, as documented on the ADNI website. All participants gave written, informed consent. The sponsors for ADNI are listed in the Acknowledgments. All subjects enrolled in this study were required to have 3.0-Tesla resting-state fMRI and T1-weighted structural MRI data available, and diagnosed as CN or MCI at the time of the imaging visit. Two hundred and seventy-seven fMRI sessions from 163 cognitively normal individuals (74.7 ± 6.3 y) and 309 fMRI sessions from 139 participants with MCI (73.4 ± 7.8 y) were included in our analysis based on the inclusion criteria.

### MR Image Acquisition and Analysis

The T1-weighted magnetization-prepared rapid acquisition gradient-echo MR images were collected with a 24 cm field of view and a resolution of 256 × 256 × 170 to yield a 1 × 1 × 1.2 mm^3^ voxel size. The resting-state fMRI data were acquired from echo-planar imaging (EPI) sequence with TR/TE = 3,000/30 ms, flip angle = 80°, 48 slices, spatial resolution = 3.3 × 3.3 × 3.3 mm^3^ and imaging matrix = 64 × 64. The raw fMRI data were first processed with slice-timing correction and rigid-body realignment of all fMRI volumes to mean fMRI volumes using SPM12 (https://www.fil.ion.ucl.ac.uk/spm/). The first five volumes of fMRI data were discarded to avoid data with unsaturated T1 signals. The mean fMRI volumes were co-registered to the native T1 structural image and the T1 image was spatially normalized to MNI152 standard space. The transformation information from co-registration and space normalization steps were applied on each fMRI volume separately to transform fMRI data to the template space. Instead of using traditional nuisance regression techniques to de-noise fMRI data, an artificial intelligence technique was applied to remove the noise in each fMRI session separately (19). This pipeline was conducted without any demographical/diagnostic information about the subject, thus these data do not bias the post-processing analysis. Previous studies (12, 19) demonstrated the improved statistical power of this technique over traditional de-noising strategies in identifying disrupted brain topology in subjects with AD.

Ninety-four cortical and subcortical regions in the cerebrum from the revised automated anatomical labeling (AAL) atlas (20) were used in our analysis. The regional time series was defined as the mean time series over gray matter voxels in each region. We then calculated Pearson's correlation between regions to measure the functional connectivity strength followed by Fisher r-to-z transformation. The connections with missing values were replaced with the mean values over all participants. The weighted functional connectivity maps were then thresholded with sparsity level varying from 0.05 to 0.5 with increment of 0.01. Caudate nodal strength was computed by first summing caudate connectivity with all the other regions at each sparsity level and then integrating over all sparsity levels to derive a single scalar, which quantified the overall caudate functional connectivity strength.

### Amyloid Status

For the subset of fMRI sessions having florbetapir or Pittsburgh compound B (PIB) amyloid positron emission tomography (PET) scans available in the same visit, we extracted the composite standard uptake value ratio (SUVR) from PET scans to determine the amyloid status. The composite SUVR score was computed by following the ADNI PET analysis pipeline. The participants with composite SUVR above 1.5 in PIB PET scans or above 1.1 in florbetapir PET scans were defined as amyloid positive. The participants with amyloid burden below the threshold were labeled as amyloid negative.

### Clinical and Cognitive Measures

A battery of neuropsychological tests was administered to participants at each visit. The cognitive measures compiled in this study included the Alzheimer's Disease Assessment Scale–Cognitive subscale (ADAS-Cog, 85-point scale), clinical dementia rating-sum of boxes (CDR-SB), Montreal Cognitive Assessment (MoCA), Trail Making Test-B (TRABSCOR), and Rey Auditory Verbal Learning Test (RAVLT) for learning and immediate recall assessment.

### Subject Characteristics

The summary of demographic characteristics of the ADNI sample was listed in Table 1. Age, ADAS-Cog, and brain amyloid status were summarized over MRI sessions; handedness, education and APOE4 genotype were summarized over subjects. Two-sample *t*-test was applied to calculate *p*-values for the difference in age, education and ADAS-Cog between women and men. For binary characteristics such as handedness, APOE4 genotype and amyloid status, the chi-squared test was conducted. Seventy one out of 146 fMRI sessions from women with MCI had amyloid PET scans available at the same visit of MRI scans (42 amyloid positive and 29 amyloid negative); 85 out of 163 fMRI sessions from men with MCI had amyloid PET scans available (42 amyloid positive and 43 amyloid negative). There was no difference between women and men in terms of handedness, APOE4 genotype and amyloid status. Women with MCI were slightly younger and had better ADAS-Cog scores than men with MCI (*p* < 0.05). Men with MCI had higher education levels than women with MCI (*p* < 0.05). Similar differences between women and men were observed in CN group. Note that instead of direct group comparisons between women and men, this study focused on how sex mediates the association between age and brain function, thus the differences in these measures do not directly bias our analyses.

**Table 1:** Demographic characteristics of study sample.

**Subjects (*n* = 302)**	**MCI (*****n*** **=** **139)**			**CN (*****n*** **=** **163)**		
	**Women (*n* = 62)**	**Men (*n* = 77)**	***p*-value**	**Women (*n* = 88)**	**Men (*n* = 75)**	***p*-value**
fMRI sessions	146 (25/8/15/10/4)	163 (30/17/21/9/0)	NA	154 (55/12/11/8/2)	123 (50/11/8/3/3)	NA
Age (y)	72.1 ± 7.5	74.9 ± 7.9	0.0354	74.1 ± 6.0	75.4 ± 6.7	0.0901
Handedness (R/L)*	59/3	70/7	0.3348	77/11	68/7	0.5203
Education (y)	15.5 ± 2.8	16.5 ± 3.0	0.0462	16.3 ± 2.5	17.0 ± 2.2	0.0616
APOE4 (+/-)*	26/36	27/50	0.4071	29/59	20/55	0.3829
ADAS-Cog	9.1 ± 4.6	10.6 ± 5.7	0.0120	5.9 ± 2.7	7.2 ± 3.6	0.0007
Amyloid positivity for individuals (+/-/NA)	32/24/6	36/37/4	NA	33/44/11	24/44/7	NA
Amyloid PET scans (+/-)^ 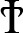 *^	42/29	42/43	0.2241	39/59	31/54	0.6443

*Age, ADAS-Cog, and amyloid PET scans are summarized over MRI sessions; handedness, education, APOE4 genotype are summarized over subjects (“-” indicates no e4 allele, “+” indicates at least one e4 allele). 2-sample t-test was carried out if not specified. One subject could have multiple amyloid PET scans, thus the number of PET scans could be more than the number of subjects. The numbers in the bracket for fMRI sessions indicate the number of individuals having 1/2/3/4/5 fMRI sessions, with average time gap of longitudinal fMRI sessions as 11 months. The amyloid positivity for each individual is based on the latest amyloid PET scan.*

*
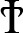
Not every fMRI sessions have corresponding amyloid PET scans available in the same visit.*

*p-values obtained by performing χ^2^-test.

### Statistical Analysis

We first evaluated the associations between the nodal strength of bilateral caudate nuclei with age separately for CN subjects and subjects with MCI, with both women and men included. Linear mixed effect (LME) model was utilized to assess the association between caudate nodal strength and age, where the intra-subject variance was modeled as a random effect grouped by individual subject and the confounding variables such as handedness, sex, education and Apolipoprotein E4 (APOE4, 0: no e4 allele, 1: at least one e4 allele) genotype were modeled as fixed effects together with age.

With the observation that the MCI group had significantly stronger connectivity associated with age than the CN group (see result), the rest of the analysis was focused on MCI group. We then included the sex-by-age interaction term in LME model to assess if the aging effect on caudate nodal strength is modulated by sex. Significant sex-by-age interaction was found in MCI but not in CN participants (see Results). We then used the LME model as described above to test the association between caudate nodal strength and age for women and men with MCI separately, except sex was no longer included in the model because it is a constant variable. From the sex-specific LME model, we extracted the adjusted caudate nodal strength after correcting for the influence of intra-subject effects and confounding factors (age is of interest and not corrected).

We further stratified the association analysis with amyloid status as noted on amyloid PET scans in a subset of fMRI data. Analysis of covariance (ANCOVA) was conducted on this subset to evaluate whether brain amyloid or sex-by-amyloid interactions modulated aging effects on caudate nodal strength. Besides assessing the influence of brain amyloid burden, we also examined the potential contribution of vascular disease, depression and anxiety by conducting the association analysis of caudate nodal strength with white matter hyperintensity (WMH) volume, geriatric depression scale (GDS) and Neuropsychiatric Inventory Questionnaire (NPI-Q) respectively among the subjects who have these scores available at the time the ADNI database was queried.

The same sex-specific LME model was then applied to investigate age-related effects on each region's connectivity with caudate nuclei (referred to as regional analysis below). Considering that nodal strength is a scalar metric for measuring overall caudate functional connectivity with all regions, regional analysis can help identify which regions make the greatest contribution to the aging effect on caudate nodal strength.

ANCOVA was conducted to investigate the role of sex in the association of age and adjusted caudate nodal strength with neuropsychological scores. Pearson's correlation was used in the *post-hoc* analysis to compute the pairwise association between age, neuropsychological scores and caudate nodal strength. With the subset of fMRI data having amyloid status available, ANCOVA was applied to evaluate the influence of sex, amyloid status and their interaction on the association of age and caudate nodal strength with neuropsychological scores.

### Independent Replication Sample

To further confirm the sex-dependent pathological aging effects observed with ADNI cohort, we have conducted the association analysis of age with caudate nodal strength with an independent sample from Center for Neurodegeneration and Translational Neuroscience (CNTN, https://www.nevadacntn.org/) cohort (21). The study was approved by the local Institutional Review Board and all participants gave written, informed consent. The MRI data in this cohort were collected at a 3.0-Tesla Siemens (Siemens Healthcare, Erlangen, Germany) Skyra scanner. The subjects clinically diagnosed as MCI were included in our analysis. The T1-weighted magnetization-prepared rapid acquisition gradient-echo MR images were collected using the GRAPPA parallel imaging technique (a factor of 2) with a 25.6 cm field of view and a resolution of 256 × 256 × 176 to yield a 1 × 1 × 1 mm^3^ voxel size. The resting-state fMRI data were acquired from accelerated echo-planar imaging sequence with a multiband acceleration factor of 8, in total 850 volumes, TR/TE = 700/28.4 ms, flip angle = 42°, 64 slices, spatial resolution = 2.3 × 2.3 × 2.3 mm^3^ and imaging matrix = 128 × 96. In the CNTN cohort, 65 participants with MCI (41 men/24 women) were included in the analysis. There were 83 fMRI sessions from 41 men with MCI (75.8 ± 6.7 y) and 51 fMRI from 24 women with MCI (72.3 ± 5.1 y) available in this cohort. The same preprocessing pipeline as with ADNI data were carried out except the fMRI denoising strategy. Because the artificial intelligence denoising technique (19) were extensively assessed only with conventional fMRI data but not with accelerated fMRI data, nuisance regression (22) were applied for the CNTN data. The same association analysis between age and caudate nodal strength as with ADNI data were carried out in this independent replication sample.

## Results

### Stronger Age-Related Increase of Caudate Nodal Strength in MCI Compared to CN

**Figure 1:**
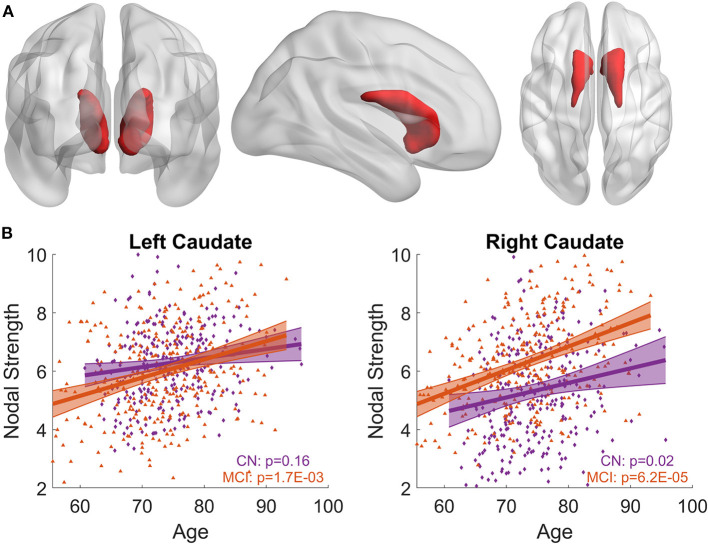
Associations between bilateral caudate nodal strength and age. **(A)** Priori-defined bilateral caudate used in the analysis. **(B)** Scatter plot of the nodal strength from left/right caudate with age for MCI and CN separately. Shaded area represents 95% confidence interval of the fitting curve. The nodal strength shown in the figure has been adjusted to remove the influence of confounding factors using LME model; see Methods section for detail. Stronger age-related increase of caudate nodal strength is observed in MCI compared to CN.

LME models relating the nodal strength of *a priori*-defined bilateral caudate nuclei (Figure 1A) to age were carried out for MCI and CN separately, with both women and men included. In CN group, bilateral caudate nuclei showed positive associations between nodal strength and age, but the association for the left caudate nucleus did not reach statistical significance (Left: *p* = 0.16; right: *p* = 0.02, see Figure 1B). The MCI group showed stronger associations with age than the CN group for both left and right caudate nuclei (Left: *p* = 0.0017; right: *p* = 6.2 × 10^−5^, see Figure 1B). When we included participants with CN and MCI in a single LME model to assess the interaction effects of age with diagnosis, significant interaction effects (Left: *p* = 0.0011; right: *p* = 0.0036) were observed when only women were considered in the model, no significant interaction effects were observed when both women and men were included or only men were included (*p* > 0.05).

### Sex Modulates Aging Effects on Caudate Nodal Strength in the MCI Group

**Figure 2:**
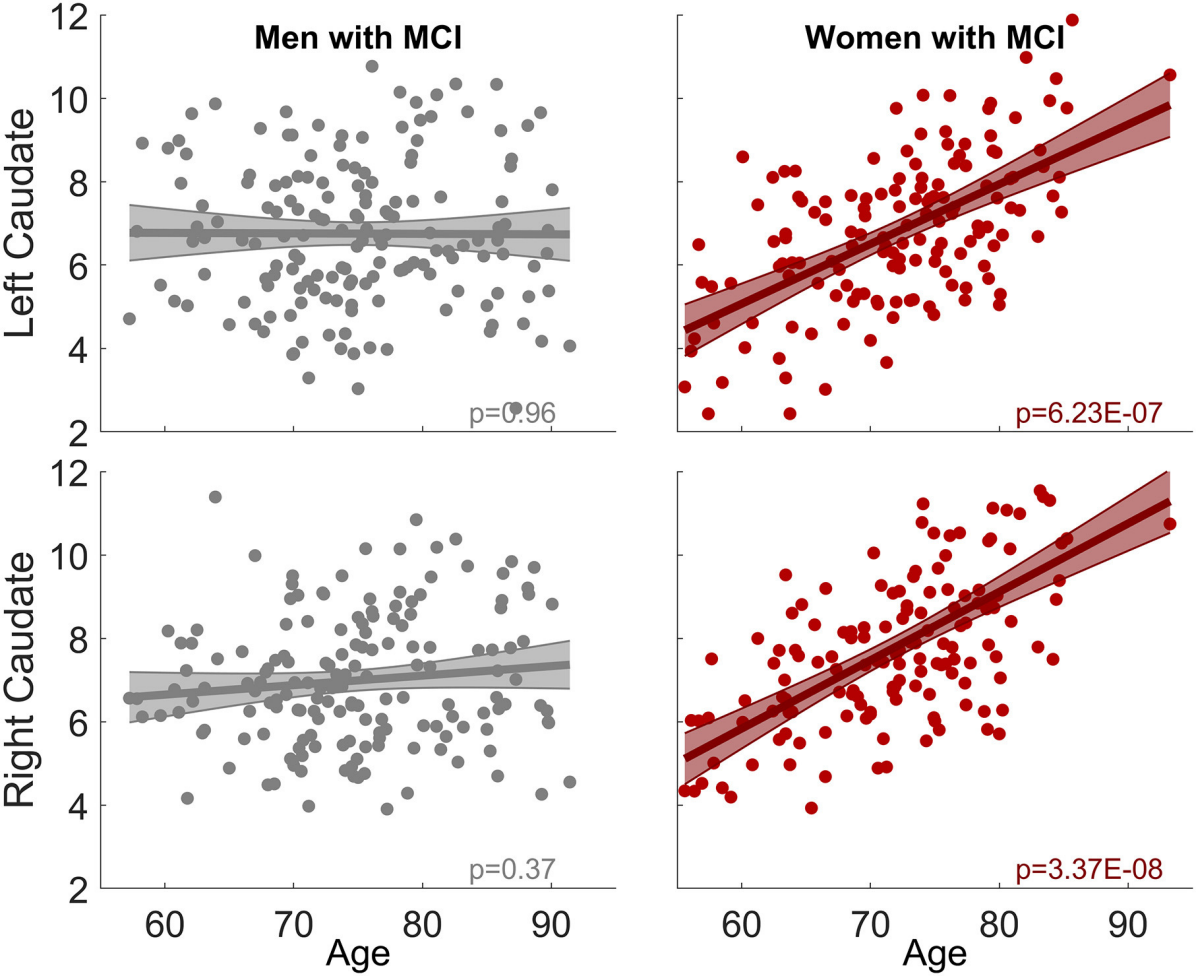
Sex-dependent association between caudate nodal strength and age among MCI group. No association is observed in men for both right and left caudate. In contrast, significant positive association is observed in women (left: *p* = 6.23 × 10^−7^, right: *p* = 3.37 × 10^−8^). The same analysis among CN group is shown in Supplementary Figure 1.

When the sex-by-age interaction term was included in LME models, there was no sex-by-age interaction effect in CN (Left: *p* = 0.66; right: *p* = 0.60). In contrast, significant sex-by-age interaction effect was found in MCI (Left: *p* = 4.6 × 10^−5^; right: *p* = 3.8 × 10^−4^), suggesting that women and men exhibit significantly different aging effects on caudate nodal strength. We split MCI into two groups based on sex and then carried out the analysis relating caudate nodal strength to age for women and men separately. Neither the right nor left caudate showed significant associations with age in men with MCI (*p* > 0.3, Figure 2). In contrast, age was significantly related to increased caudate nodal strength in women with MCI (Left: *p* = 6.23 × 10^−7^; right: *p* = 3.37 × 10^−8^). Sex-specific analysis in CN participants showed that caudate nodal strength did not associate with age in either women or men (*p* > 0.05; see Supplementary Figure 1). The significance of aging effect on nodal strength of all ROIs in CN and MCI group were shown in Supplementary Figure 2 for women and men separately.

### Sex-Dependent Association Stratified With Amyloid Status

**Figure 3:**
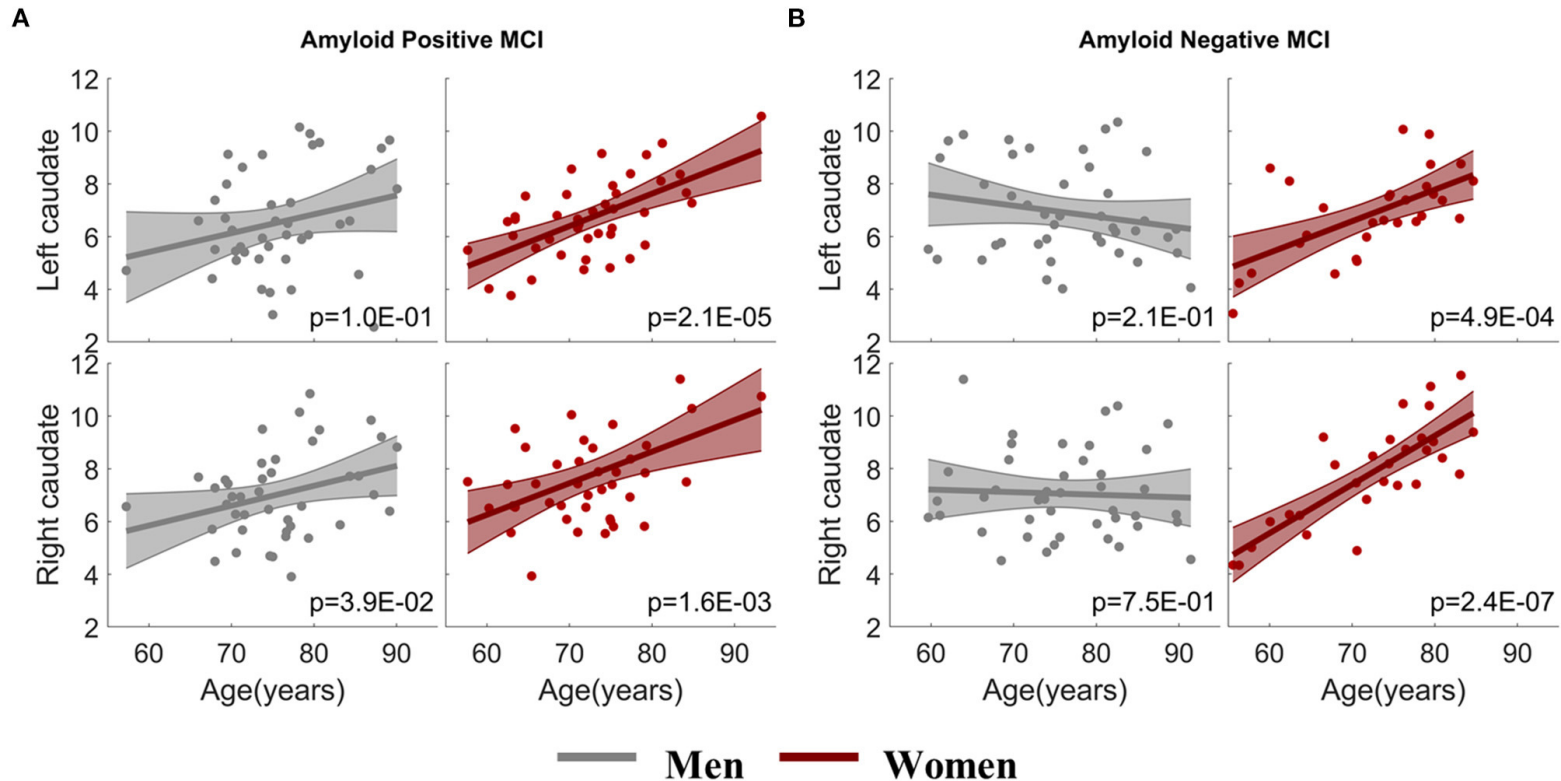
Sex-dependent association analysis between caudate nodal strength and age with stratified amyloid status. **(A)** Association analysis within amyloid positive MCI participants. **(B)** Association analysis within amyloid negative MCI participants. The x-axis is age and the y-axis is the corrected caudate nodal strength after adjusting the influence of confounding factors.

To address whether brain amyloid could make a difference on the aging effects observed in the MCI group, we assessed the association between caudate nodal strength and age with stratified amyloid status in the fMRI data subset having amyloid PET available in the same visit. One hundred and fifty-six fMRI sessions from the MCI group had the corresponding amyloid PET scans in the same visit, 72 sessions (29 W/ 43 M) were identified as amyloid negative and 84 sessions (42 W/ 42 M) identified as amyloid positive (Table 1). ANCOVA showed that aging effects on caudate nucleus were modulated by sex as expected (*p* < 0.05), but not modulated by sex-by-amyloid interactions or solely by amyloid (*p* > 0.05). In the *post-hoc* analysis, bilateral caudate nodal strength in both amyloid positive and negative woman participants with MCI had significant associations between age and bilateral caudate nodal strength (Figure 3; amyloid positive: left *p* = 2.1 × 10^−5^, right *p* = 1.6 × 10^−3^; amyloid negative: left *p* = 4.9 × 10^−4^, right *p* = 2.4 × 10^−7^). Right caudate nodal strength in amyloid positive men with MCI weakly correlated with age (*p* = 0.039) but not for left caudate. No aging effect was observed in amyloid negative men with MCI. Overall, consistent sex-dependent aging effects were observed in amyloid positive and amyloid negative MCI participants. In terms of the relevance of white matter hyperintensity, GDS and NPI-Q to caudate nodal strength, these factors were weakly associated or not associated with caudate nodal strength in both women and men with MCI (Supplementary Figure 3).

### Regional Heterogeneity Contributes to Sex-Dependent Aging Effects on Caudate Nodal Strength

**Figure 4:**
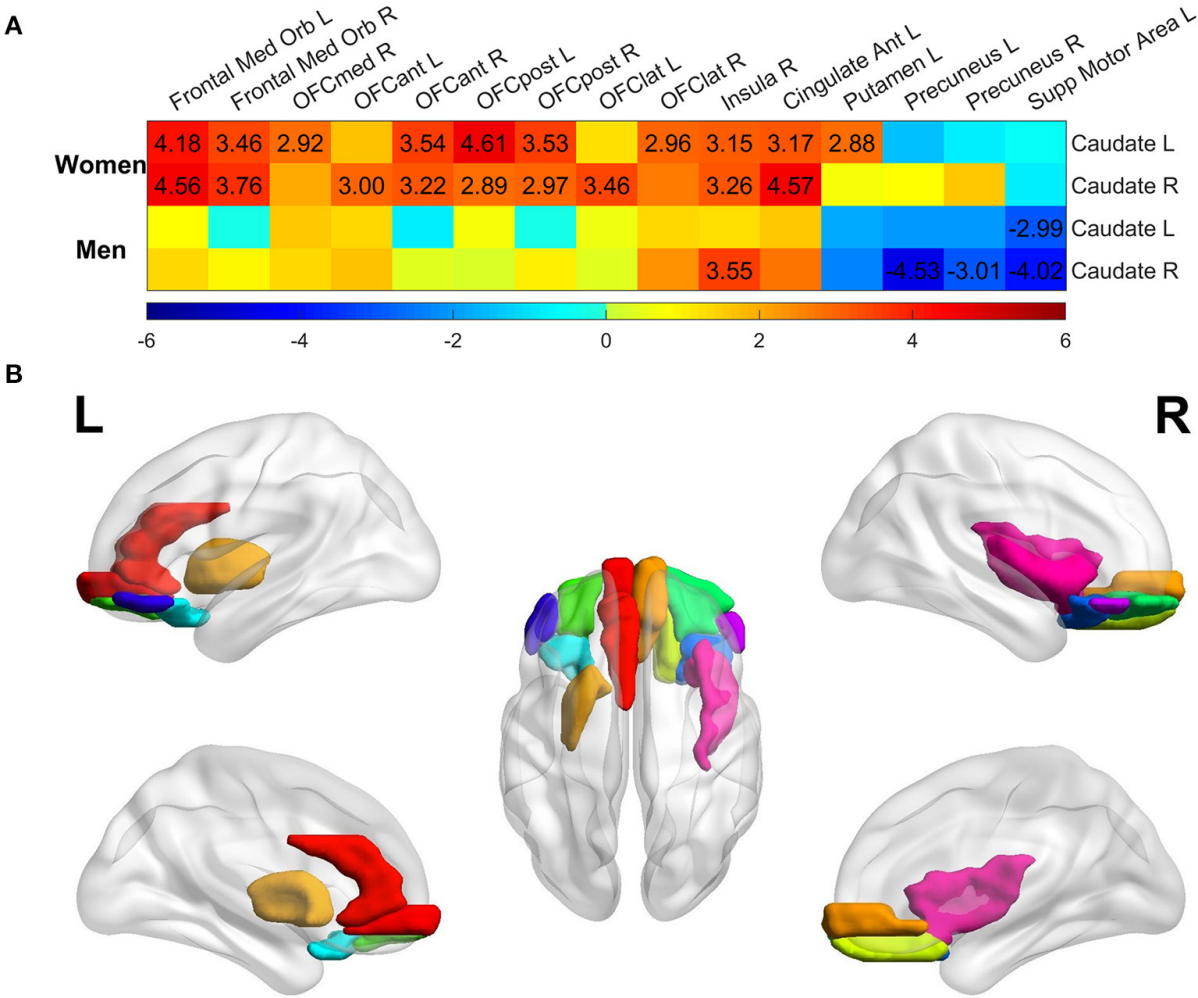
Regional analysis of aging effect on functional connectivity of individual ROI with left/right caudate among the MCI group. **(A)** *t*-statistical values of aging effect on functional connectivity of individual region with left/right caudate. All regions were assessed, but only regions having association over the significance threshold *p* = 0.005 in at least one scenario were listed in the figure. Only the *t*-values with significance level *p* < 0.005 were marked in the plot. **(B)** Brain regions having significant association with age thresholded at *p* < 0.005 in women with MCI, regardless of the connectivity with left or right caudate.

We next assessed age-related effects on each region's connectivity with caudate nucleus, for women and men with MCI separately, to examine which region contributes the most to the aging effects on caudate nodal strength. The t-statistic map of aging effects on caudate-region connectivity was shown in Figure 4A. Only the brain regions having associations over the significance level *p* < 0.005 (uncorrected for multiple comparison; t values marked in the figure) at least in one scenario (connectivity with either left or right caudate nucleus in women or men with MCI) were listed. A stricter significance level was used to reduce the potential false association with multiple testing. Regional analyses revealed that the positive association of caudate nodal strength with age in women was driven mainly by the connectivity with the orbital gyrus (including medial, anterior, lateral, and posterior), bilateral medial orbitofrontal gyrus, right insula, left anterior cingulate cortex and left putamen. The majority of these regions showed positive associations in men with MCI, but the associations did not reach the specified statistical significance level except for those of the right insula. Women did not have any region showing negative associations with age (*p* > 0.005). In contrast, negative associations were observed in men in bilateral precuneus and left supplementary motor area. Left and right caudate nucleus showed similar spatial pattern in the association analysis of age with caudate-region connectivity, with no hemispheric dominance observed. The brain regions having strong associations (*p* < 0.005) with age in women with MCI are shown in Figure 4B, regardless of whether the connectivity is with left or right caudate nucleus.

### Sex-Dependent Association Between Caudate Nodal Strength, Age, and Cognitive Measures in MCI

**Figure 5:**
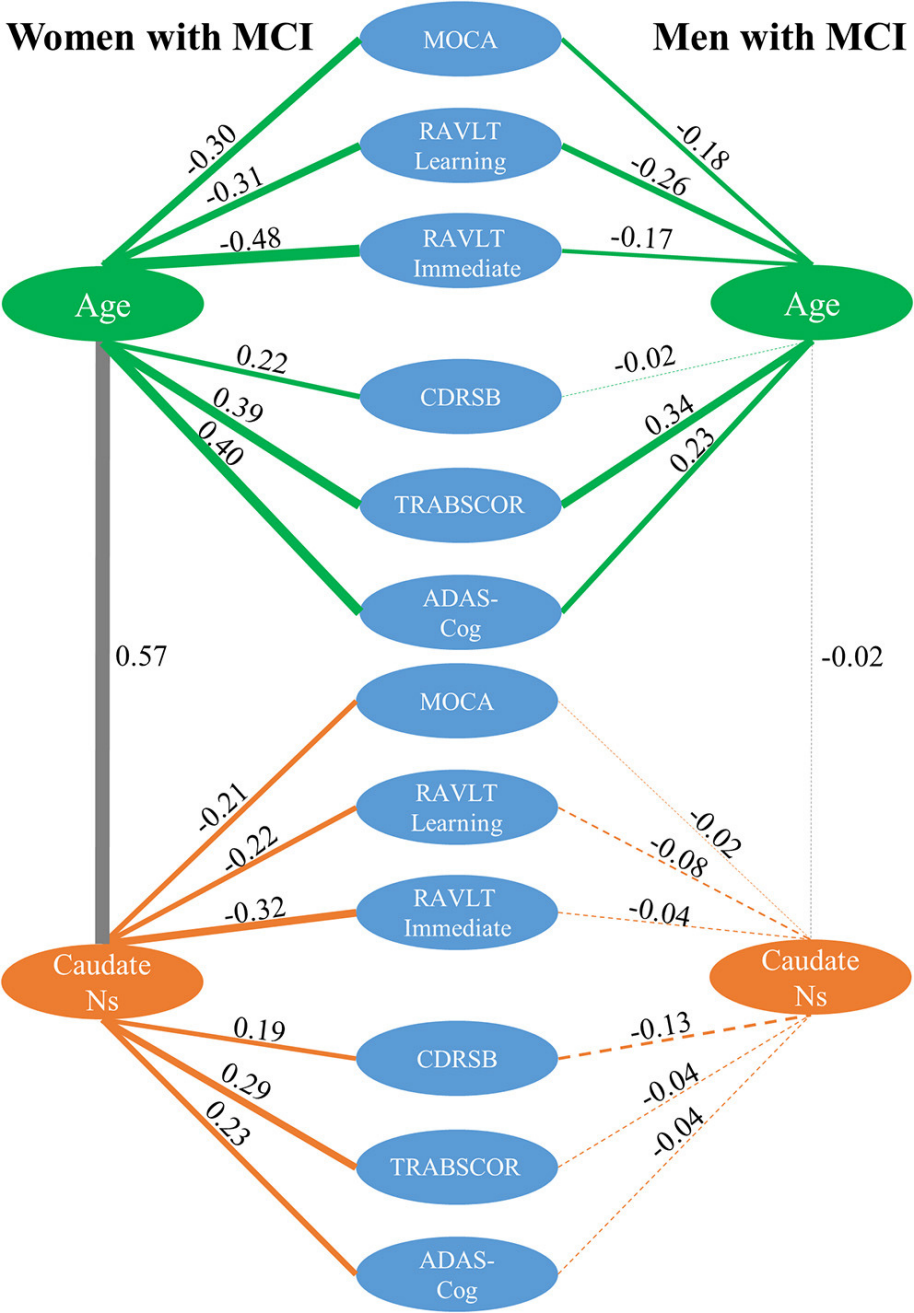
Correlation analysis among caudate nodal strength (Ns), neuropsychological scores, and age for women and men with MCI separately. For simplicity, only left caudate nodal strength was presented in the figure. Green, gray, and orange lines represent the correlation between age and neuropsychological scores, between age and caudate nodal strength, and between caudate nodal strength and neuropsychological scores, respectively. Pearson's correlations are marked in the figure with line thickness proportional to the correlation strength. A solid line means the correlation is significant (*p* < 0.05) and dashed line means the correlation is not significant (*p* > 0.05).

ANCOVA showed that the association of age with neuropsychological measures in MCI were not modulated by sex except for RAVLT immediate (*p* = 0.003) and CDR-SB (*p* = 0.04) scores (see the second column in Table 2). Older age was significantly associated with worse cognitive performance across six neuropsychological measures in both women and men with MCI, except for CDR-SB in men with MCI, as shown in the top panel of Figure 5. Caudate nodal strength was strongly related to age in women with MCI with Pearson's correlation of 0.57, which means more than 30% of the variance of caudate nodal strength in women with MCI could be explained by aging effects. In contrast, aging effects explain little of the variance of caudate nodal strength in men with MCI (<1%).

**Table 2:** Significance of sex in the association between neuropsychological scores and age/caudate nodal strength.

***p*-value (uncorrected; significance of sex-dependency in the association)**	**Age**	**Caudate nodal strength**
MOCA	0.198	0.091
RAVLT learning	0.198	0.219
RAVLT immediate	**0.003**	**0.009**
CDR-SB	**0.036**	**0.005**
TRABSCOR	0.724	**0.003**
ADAS-Cog	0.143	**0.010**

*The p-values noted in the table were derived from analysis of covariance. Significant sex-dependent associations were observed for the association between caudate and neuropsychological measures except for MOCA (trend toward significance) and RAVLT learning. Significant sex-dependent association with age is observed for RAVLT immediate and CDR-SB scores (marked in bold)*.

Women and men overall had significantly different associations between caudate nodal strength and neuropsychological measures except for RAVLT learning and MoCA (see the third column in Table 2). Higher caudate nodal strength was related to worse neuropsychological scores in women with MCI (MOCA *r* = −0.21; RAVLT learning *r* = −0.22; RAVLT immediate *r* = −0.32; CDR-SB *r* = 0.19; TRABSCOR *r* = 0.29; ADAS-Cog *r* = 0.23). In contrast, caudate nodal strength in men with MCI was neither associated with age nor neuropsychological measures (*p* > 0.05).

When ANCOVA was applied on the subset of fMRI data having amyloid PET available, amyloid status significantly modulated the association of age with multiple neuropsychological scores, including MOCA (*p* = 0.01), ADAS-Cog (*p* = 0.01), and RAVLT learning score (*p* = 0.004). Significant sex-by-amyloid interaction effects were observed in the association of age with RAVLT learning score (*p* = 0.01). The association of age with other neuropsychological scores were not modulated by amyloid status. Neither amyloid status nor sex-by-amyloid interaction were observed to mediate the association of caudate nodal strength with neuropsychological scores. The pair-wise associations among age, neuropsychological scores and caudate nodal strength were shown separately for amyloid positive and negative participants with MCI in Supplementary Figure 4.

### Consistent Sex-Dependent Aging Effects on Caudate Nodal Strength Observed With Replication Sample

We conducted the association of age with caudate nodal strength for women and men with MCI separately in the CNTN cohort. Strong positive Pearson's correlation (*r*) between age and adjusted caudate nodal strength were observed in women with MCI but not in men with MCI (Supplementary Figure 5, women: left caudate *r* = 0.55, right caudate *r* = 0.48; men: left caudate *r* = −0.17, right caudate *r* = −0.11). Consistent with ADNI cohort, only women with MCI had significant association of age with caudate nodal strength (women: left caudate *p* = 3.4 × 10^−5^, right caudate *p* = 3.9 × 10^−4^; men: left caudate *p* = 0.14, right caudate *p* = 0.35). Women with CN and Men with MCI or CN did not show the association.

## Discussion

In the current study, sex is demonstrated to modulate aging effects on caudate nodal strength among MCI participants but not in CN participants. A strong positive associations of age with bilateral caudate nodal strength exists only in women with MCI but not in men with MCI. For both amyloid positive and negative MCI participants, similar sex-dependent aging effects are observed. The connectivity between caudate nucleus and ventral prefrontal cortex substantially contributes to the aging effects on overall caudate connectivity (characterized by nodal strength) in women with MCI. Caudate nodal strength in men with MCI does not correlate with age. Even though older age was associated with multiple cognitive measures in both women and men with MCI, only women with MCI demonstrated that higher caudate nodal strength was closely related to worse cognition, suggesting that caudate connectivity may provide a sensitive imaging biomarker of pathological aging effects in women with MCI.

Multiple prior studies found prominent age-related brain functional and structural alterations of the caudate nucleus (5, 23–25), with the majority of findings observed by comparing the difference between old and young healthy adults, suggesting that the caudate nucleus is vulnerable to aging effects even in the absence of disease. Our association analysis suggests that the normal aging process has a subtle effect on caudate nodal strength in the cognitively normal older population. The aging effects on caudate function could be related to a variety of biological alterations occurring in the caudate, such as the loss of physiological asymmetry in dopamine transmission during normal aging (26). In addition, age-related effects on caudate function could be related to cognitive decline in the normal aging process, studies with both healthy subjects and disease populations demonstrate the involvement of caudate nucleus in cognition (27–29).

While age-related change in the caudate is recognized as an important factor to predict cognitive decline over the life span (28), compared to the medial temporal lobe system, far less attention has been paid to the involvement of the caudate in dementia, and most of these prior MRI studies focused on volumetric changes of caudate nucleus with diverse conclusions (30–32). Our association analysis revealed that aging effects on caudate function in MCI is modulated by sex. Stronger aging effects on caudate nodal strength were observed in women with MCI but not in men with MCI. A previous fMRI study showed differing brain functional alteration between MCI and CN in women and men, based on multiple global network metrics (12). These observations together suggest that sex plays an important role in modulating brain functional topology. Sex differences in dopaminergic signaling have been linked to sex-dependent heterogeneity in various neuropsychiatric disorders (33). The polymorphisms of the DRD2/ANKK1 gene, a gene linked to striatal dopamine functions, showed their relevance to caudate blood oxygen level-dependent (BOLD) fMRI activation and neurocognitive deficits (34). Considering that caudate nucleus is one critical region in the dopamine pathway, these studies collectively suggest that the sex-dependent effect on caudate in MCI may be related to sex-dependent heterogeneity of dopaminergic signaling, providing indirect evidence to suggest that dopaminergic dysfunction has a pathogenic role in cognitive decline symptoms of AD (35). Multimodal studies with both fMRI signal and dopamine available would be helpful to further evaluate the role of dopamine on sex-dependent aging effects in caudate.

Independent from the main ADNI cohort used in the study, similar sex-dependent aging effects on caudate nodal strength in MCI group were reproduced with the CNTN cohort. In this sample, a significant positive association between age and caudate nodal strength were observed only in women with MCI. Collectively, similar sex-dependent association were found in the data collected with both conventional and fast fMRI sequence from two independent studies, demonstrating the robustness and reproducibility of the finding.

Putamen and caudate nucleus together form the dorsal striatum; they are the primary input nuclei of basal ganglia, receiving inputs from wide regions of cortex. An influential model linking basal ganglia to cortex demonstrated that striatum is involved in at least five functionally segregated corticostriatal circuits (27, 36), including motor, oculomotor, dorsolateral, ventral/orbital and anterior cingulate circuits. The association analysis between age and connectivity of individual regions with the caudate nucleus revealed that the connectivity of caudate nucleus with regions in ventral prefrontal cortex substantially contributed to the aging effect on caudate connectivity, suggesting that aging effects on caudate nucleus in women with MCI may be relevant to ventral/orbital and anterior cingulate circuits. Increased fMRI activation in prefrontal cortex in healthy older adults compared to younger adults is observed on a variety of cognitive tasks, which is widely hypothesized as prefrontal cortex compensating for the failing neural function in other brain regions, such as hippocampus (37, 38). Increased connectivity between ventral prefrontal cortex and caudate nucleus with age, particularly in women with MCI, may play a role in the compensatory mechanism. Age-related reduced connectivity of supplementary motor area and precuneus with caudate nucleus in men with MCI may be related to the re-organized motor circuit (39). The distinct directionalities of aging effects between women and men underscore sex as a key demographic factor for assessing whether higher or lower caudate connectivity is beneficial in MCI. The discrepancy of brain regions involved in aging effects on caudate nucleus suggest that distinct brain regions should be targeted when developing or assessing therapy to delay or prevent progression of MCI to dementia.

Considering the influence of brain amyloid in MCI group (40, 41), we hypothesized that amyloid positive or negative MCI would exhibit distinct aging effects. Contrary to expectations, caudate nodal strength was consistently demonstrated to be strongly positively associated with age in women with MCI, regardless of the amyloid status. As to men with MCI, only right caudate nodal strength in amyloid positive participants weakly associated with age; bilateral caudate nuclei in amyloid negative participants and left caudate nucleus in amyloid positive participants did not show aging effect. Similarly, atrophy of the caudate nucleus is neither associated with amyloid nor implicated in the subsequent development of AD dementia (25). Even though amyloid status mediated the association of age with multiple neuropsychological scores, amyloid pathology may not be the critical factor contributing to the aging effects on caudate connectivity in women with MCI. Neither amyloid status nor sex-by-amyloid interaction mediated the association of caudate nodal strength with neuropsychological scores. In addition, based on the association analysis with WMH, GDS and NPI-Q, vascular diseases, depression, and anxiety were unlikely to be the main factors contributing to the sex-dependent aging effect on caudate function in MCI.

Since MCI subjects exhibit faster cognitive decline than typical of normal aging, our finding of a significant association of older age with worse neuropsychological measures is expected. The striking difference between women and men with MCI is the distinct correlation of caudate nodal strength with neuropsychological measures and age, indicating that the caudate nucleus is highly sensitive to pathological cognitive aging in women with MCI. The substantial contribution of ventral prefrontal cortex to the aging effect on caudate nucleus supports the major theory of cognitive aging, which attributes many aspects of cognitive decline to altered prefrontal cortical function (42). Although the current study is not structured to illustrate the causal role of caudate function on cognitive decline, our finding supports the hypothesis that caudate nodal strength can serve as a sensitive biomarker to facilitate detection and monitoring of brain functional alterations with aging or when assessing the efficacy of therapies.

There are a few limitations with the study. First, there is no consensus regarding the best brain parcellation scheme when performing functional connectivity analysis. We conducted the analysis with the most commonly used AAL structural atlas (20). The nodal strength characterized in the study can be affected substantially by the parcellation scheme. Atlas standardization is recommended in comparisons with other studies. Instead of using structural atlases, multiple functional atlases were proposed in the last decade based on fMRI data from several cohorts (43–45). However, the merit of functional atlases remains to be determined; even a single individual may need different functional parcellation definitions under various tasks (46). The generalizability of functional atlases from one cohort to another, which could be influenced by age and disease, also requires more validation. Second, although multiple fMRI sessions from a single individual were included in the study, because of the limited number of longitudinal fMRI scans, the analysis was conducted in a cross-sectional fashion (intra-subject effect was modeled as a random effect in the LME model). More longitudinal data from the ongoing ADNI project and other cohorts would be helpful to verify our observations. It remains to be confirmed if the alteration of caudate nodal strength is sensitive at the individual level, which is critical for its clinical application. Third, MCI is a heterogeneous disease condition, it is unclear if the sex-dependent association is partially due to some unaccounted factors biased toward women with MCI. For example, genetic risk factors beyond APOE genotype may contribute to the sex-dependent aging effects.

## Conclusion

In summary, we successfully demonstrated sex-modulated aging effects on caudate functional connectivity in the MCI group using resting-state fMRI data. A striking aging effect on bilateral caudate functional connectivity in women but not in men with MCI participants were observed in both ADNI and CNTN cohorts, without being significantly mediated by brain amyloid. Similar sex dimorphism was observed in the association analysis between caudate connectivity and a battery of neuropsychological measures. Collectively, our study shows that characterizing caudate function using resting state fMRI provide new insights into how age affects women and men differentially in MCI. Our findings could serve as a pathway for understanding sex-dependent pathological effects on brain function in neurodegenerative diseases.

## Author's Note

Part of data used in preparation of this article were obtained from the Alzheimer's Disease Neuroimaging Initiative (ADNI) database (http://adni.loni.usc.edu/). As such, the investigators within the ADNI contributed to the design and implementation of ADNI and/or provided data but did not participate in analysis or writing of this report. A complete listing of ADNI investigators can be found at http://adni.loni.usc.edu/wp-content/uploads/how_to_apply/ADNI_Authorship_List.pdf.

## Data Availability Statement

The datasets presented in this study can be found in online repositories. The names of the repository/repositories and accession number(s) can be found at: https://www.nevadacntn.org/ and http://adni.loni.usc.edu/.

## Ethics Statement

The studies involving human participants were reviewed and approved by Cleveland Clinic and ADNI sites. The patients/participants provided their written informed consent to participate in this study.

## Author Contributions

ZY and DC: drafting/revision of the manuscript for content, including medical writing for content, study concept or design, and analysis or interpretation of data. JZKC: drafting/revision of the manuscript for content, including medical writing for content and analysis or interpretation of data. JLC: drafting/revision of the manuscript for content, including medical writing for content, major role in the acquisition of data, and study concept or design. AR: drafting/revision of the manuscript for content, including medical writing for content and major role in the acquisition of data. JK: drafting/revision of the manuscript for content, including medical writing for content and analysis or interpretation of data. All authors contributed to the article and approved the submitted version.

## Funding

This research project was supported by the NIH (Grant No. 1RF1AG071566, and COBRE 5P20GM109025), Cleveland Clinic Keep Memory Alive Young Investigator Award, The Women's Alzheimer's Movement, a private grant from Stacie and Chuck Matthewson, a private grant from Peter and Angela Dal Pezzo, and a private grant from Lynn and William Weidner. Part of the data collection and sharing for this study was funded by the Alzheimer's Disease Neuroimaging Initiative (ADNI; National Institutes of Health Grant U01 AG024904) and DOD ADNI (Department of Defense award number W81XWH-12-2-0012). ADNI was funded by the National Institute on Aging, the National Institute of Biomedical Imaging and Bioengineering, and through generous contributions from the following: AbbVie, Alzheimer's Association; Alzheimer's Drug Discovery Foundation; Araclon Biotech; BioClinica, Inc.; Biogen; Bristol-Myers Squibb Company; CereSpir, Inc.; Cogstate; Eisai Inc.; Elan Pharmaceuticals, Inc.; Eli Lilly and Company; EuroImmun; F. Hoffmann-La Roche Ltd and its affiliated company Genentech, Inc.; Fujirebio; GE Healthcare; IXICO Ltd.; Janssen Alzheimer Immunotherapy Research & Development, LLC.; Johnson & Johnson Pharmaceutical Research & Development LLC.; Lumosity; Lundbeck; Merck & Co., Inc.; Meso Scale Diagnostics, LLC.; NeuroRx Research; Neurotrack Technologies; Novartis Pharmaceuticals Corporation; Pfizer Inc.; Piramal Imaging; Servier; Takeda Pharmaceutical Company; and Transition Therapeutics. The Canadian Institutes of Health Research is providing funds to support ADNI clinical sites in Canada. Private sector contributions are facilitated by the Foundation for the National Institutes of Health (www.fnih.org). Private sector funders had no role in the study design, collection, analysis, interpretation of data, the writing of this article or the decision to submit it for publication. The grantee organization is the Northern California Institute for Research and Education, and the study is coordinated by the Alzheimer's Therapeutic Research Institute at the University of Southern California. ADNI data are disseminated by the Laboratory for Neuro Imaging at the University of Southern California.

## Conflict of Interest

The authors declare that the research was conducted in the absence of any commercial or financial relationships that could be construed as a potential conflict of interest.

## Publisher's Note

All claims expressed in this article are solely those of the authors and do not necessarily represent those of their affiliated organizations, or those of the publisher, the editors and the reviewers. Any product that may be evaluated in this article, or claim that may be made by its manufacturer, is not guaranteed or endorsed by the publisher.
