# Supplementary material for: Sex Modulates the Pathological Aging Effect on Caudate Functional Connectivity in Mild Cognitive Impairment
Source: Front Psychiatry. 2022 Apr 5;13:804168. doi: 10.3389/fpsyt.2022.804168 (PMC9037326; doi:10.3389/fpsyt.2022.804168)
Supplement: Supplementary file 1 [file Data_Sheet_1.pdf]

Supplementary Material

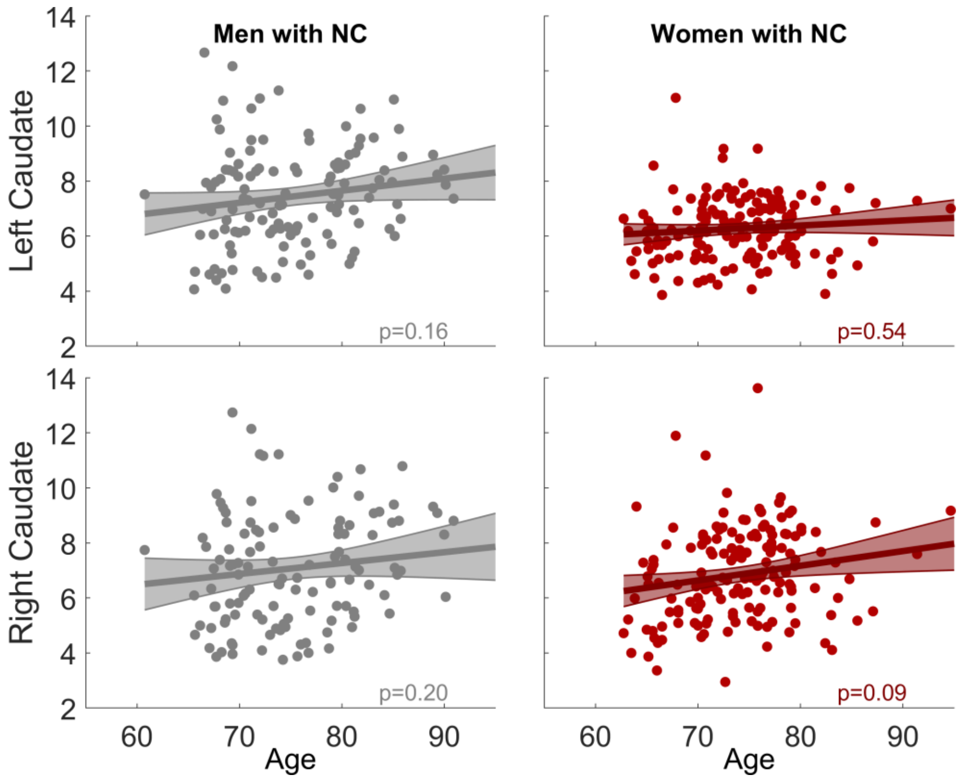

Figure S1. Scatter plot of caudate nodal strength with age for women and men separately in CN group. Sex-specific linear mixed effect (LME) model is used to derive the adjusted caudate nodal strength (y-axis) in the plot. Note that the y-axis in Figure 1 is derived from LME model with both women and men included, and sex is a confounding factor in the model.

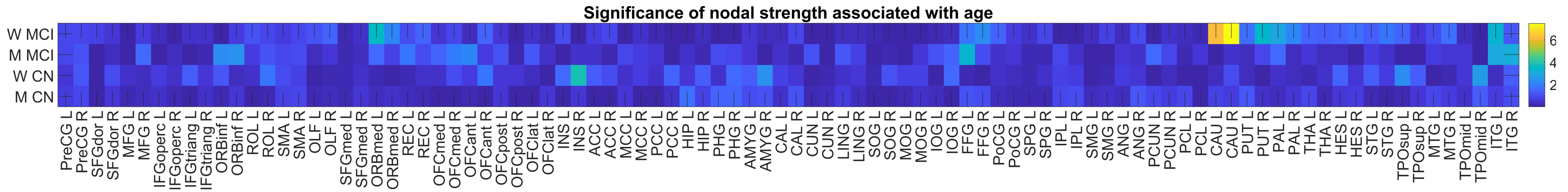

Figure S2. Significance of nodal strength association with age for women and men separately in CN and MCI groups. –log10(*p* value) is shown in the figure.

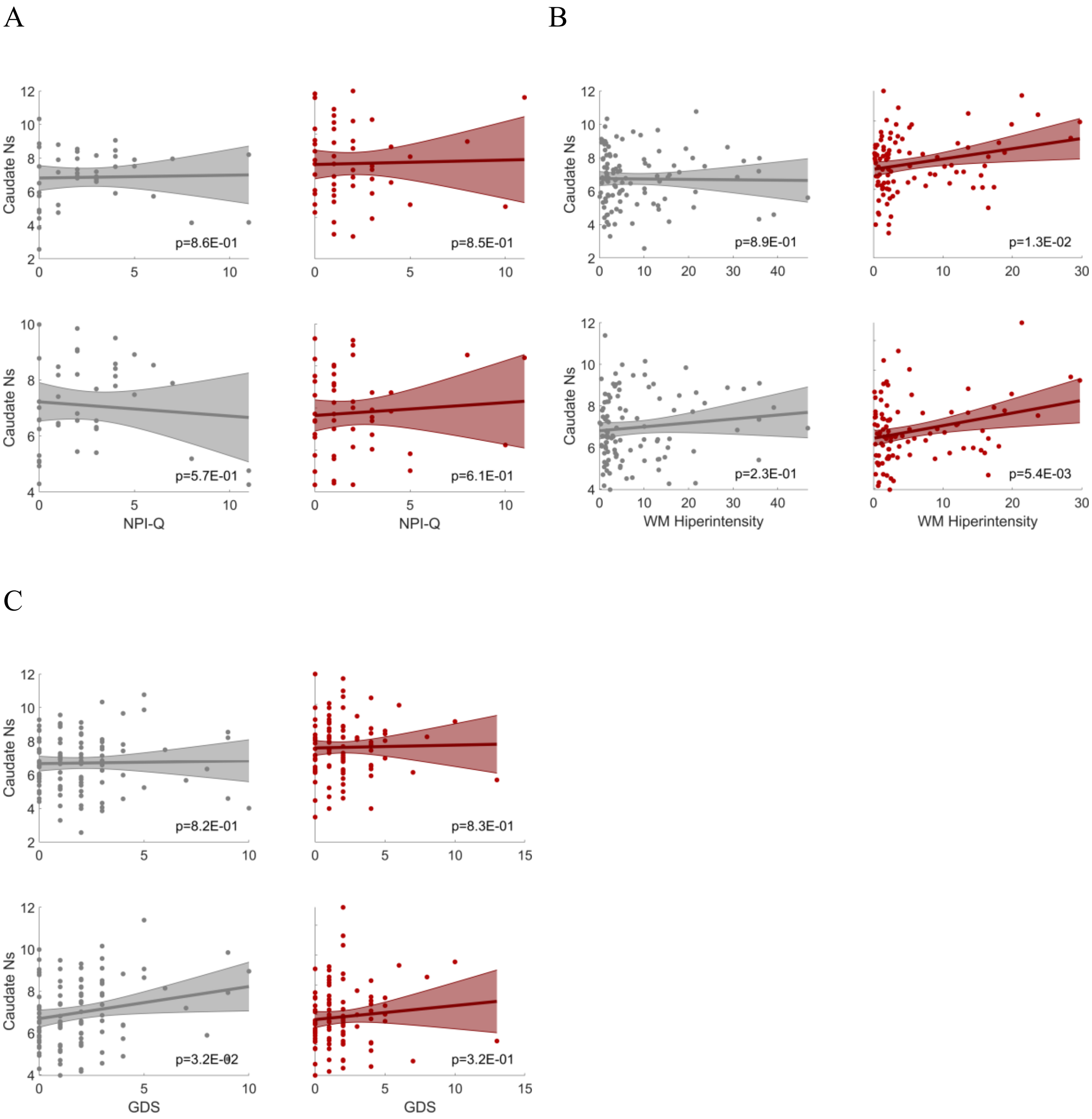

Figure S3. Association analysis of caudate nodal strength with NPI-Q (A), white matter hyperintensity (B) and GDS score. Scatter plots and the linear fitting curves with 95% confidence intervals for women (dark red) and men (gray) with MCI were drawn in the figure. For each subfigure, top panel was for left caudate and bottom panel was for right caudate.

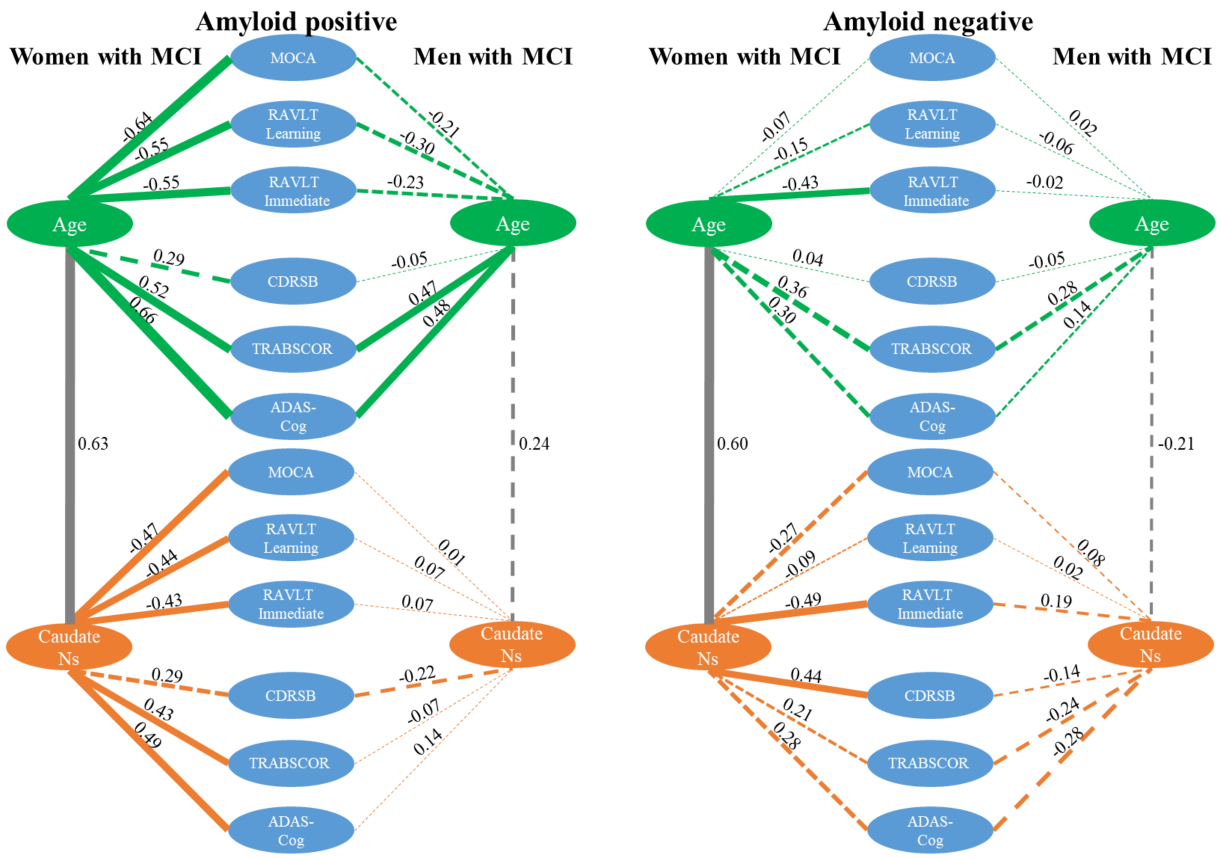

Figure S4. Associations among age, neuropsychological scores and caudate nodal strength (Ns) for amyloid positive (left) and negative (right) participants MCI separately.

A

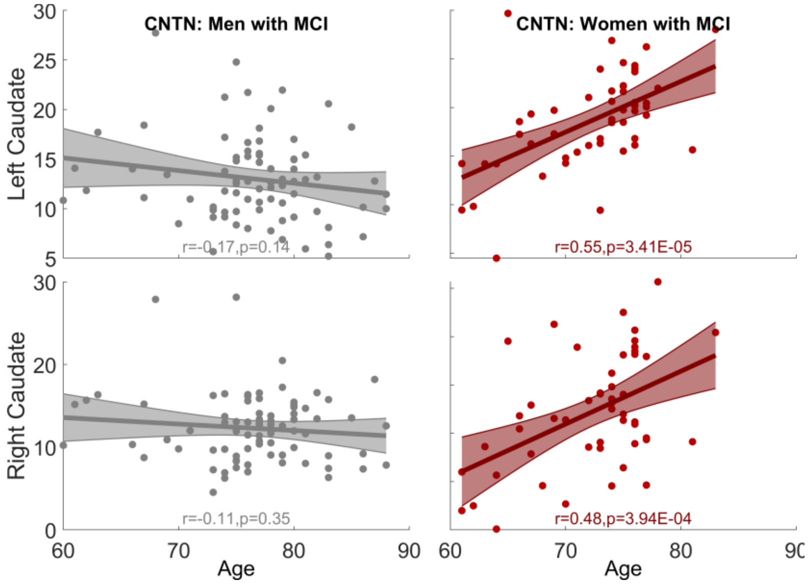

B

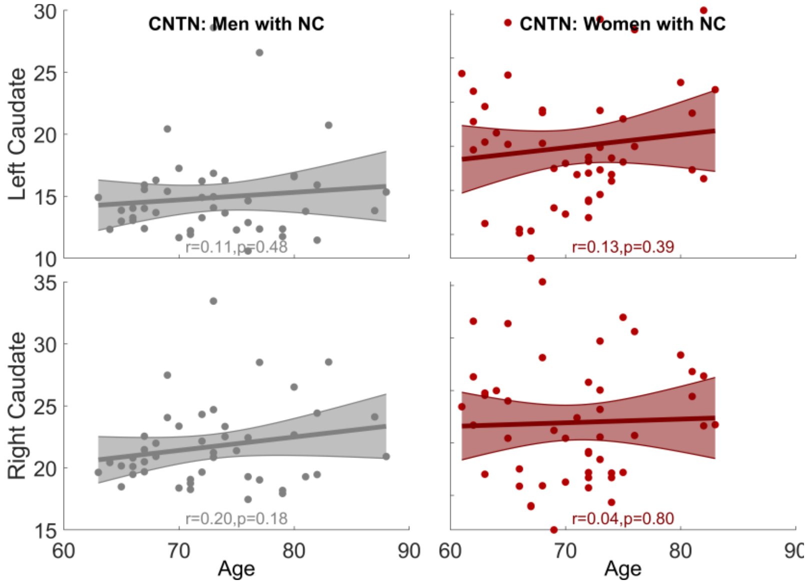

Figure S5. Sex-dependent association between caudate nodal strength and age among MCI (A) and CN (B) group from the independent CNTN cohort. In the MCI group, the Pearson's correlations (*r*) between age and adjusted left/right caudate nodal strength are 0.55/0.48 for women and -0.17/-0.11 for men. The association is only significant in women with MCI (left

caudate p=3.4x10-5, right caudate p=3.9x10-4) but not in men with MCI (left caudate p=0.14, right caudate p=0.35). The association is not observed in CN group for either women or men.
